# Grand Challenges in Microbe-Driven Marine Carbon Cycling Research

**DOI:** 10.3389/fmicb.2020.01039

**Published:** 2020-06-23

**Authors:** Hongyue Dang

**Affiliations:** State Key Laboratory of Marine Environmental Science, Institute of Marine Microbes and Ecospheres, College of Ocean and Earth Sciences, Frontiers Science Center for Ocean Carbon Sink and Climate Change, Fujian Key Laboratory of Marine Carbon Sequestration, Xiamen University, Xiamen, China

**Keywords:** biological carbon pump, blue carbon, chemolithoautotroph, climate change, climate remediation, greenhouse gases, marine carbon cycle, microbial carbon pump

Given the climate change crisis, it is urgent to reduce anthropogenic CO_2_ emissions and explore climate geoengineering opportunities. The ocean plays critical roles in global carbon cycling and may provide a solution for climate change mitigation (Buesseler et al., 2008; Yoon et al., 2018). An accurate understanding of the processes and mechanisms of the marine carbon cycle and its interactions with human-driven climate change is fundamentally important. The Aquatic Microbiology Section made important contributions of new knowledge and insights to this research field, including focused investigations on marine primary production, organic matter biodegradation and biotransformation, and microbial responses to natural and anthropogenic environmental gradients and stressors (e.g., Bullerjahn and Post, 2014; Labbate et al., 2016; Daniel et al., 2018; Gutierrez et al., 2018; Mayali, 2018; Villar-Argaiz et al., 2018; Wilson and Church, 2018; Dang et al., 2019; Murillo et al., 2019; Sala et al., 2019; Wietz et al., 2019).

## Coastal vs. Oceanic Blue Carbon Sinks

Vegetated coastal ecosystems (VCEs), including mangrove forests, salt marshes, seagrass meadows, and seaweed beds, constitute intense blue carbon sinks at the land–ocean transition zones (Nellemann et al., 2009; Macreadie et al., 2019). Conserving and restoring VCEs for maintaining and enhancing blue carbon sequestration have been proposed as an integral part of strategies for climate remediation (Geraldi et al., 2019). However, the VCEs are also hotspots of non-CO_2_ greenhouse gas emissions due to anthropogenic eutrophication-enhanced microbial activities including CH_4_ and N_2_O production (Angell et al., 2018; Dang and Li, 2018). Although VCEs may contribute to climate change mitigation at the local and national scales, they occupy a very limited spatial extent on Earth and thus their climate remediation potential is small at the global scale (Taillardat et al., 2018).

The ocean is a huge carbon sink and of enormous climate remediation potential. It absorbs atmospheric CO_2_ via both physical and biological processes. Unfortunately, excess CO_2_ absorbed via physical processes causes ocean acidification (Doney et al., 2009). Decreasing seawater pH and carbonate saturation states change the food web structure and biogeochemical cycles of the ocean, causing loss of marine ecosystem stability and services (Cooley et al., 2016; Hurd et al., 2018). Ocean acidification may even trigger a sixth mass extinction event (Veron, 2008). Therefore, it is inappropriate to inject more CO_2_ into the ocean *via* physical methods for climate remediation (IPCC, 2005; Reith et al., 2019). On the contrary, biological CO_2_ fixation converts inorganic carbon into organic matter, providing a more favorable mechanism of the ocean to absorb atmospheric CO_2_ (Falkowski and Raven, 2007; Siegel et al., 2016).

## Biological Carbon Pump vs. Microbial Carbon Pump

Marine ecosystems contribute half of all biological carbon fixation on Earth. However, in order for long-term carbon sequestration, photosynthetically fixed carbon needs to be transported to and stored in deep ocean waters and sediments. The biological carbon pump (BCP) helps fulfill this function, transporting particulate organic carbon (POC) from the ocean surface to its interior and thus contributing to climate modulation on geological time scales (Falkowski and Raven, 2007; Le Moigne, 2019). The microbial carbon pump (MCP) is another biological carbon sequestration mechanism (Jiao et al., 2010). The essence of MCP is the microbial transformation of labile dissolved organic carbon (LDOC) into recalcitrant dissolved organic carbon (RDOC) that is resistant to further biological degradation and thus maintained in the ocean for decades to millennia (Ogawa et al., 2001; Jiao et al., 2014). Both structural recalcitrance and low concentration of DOC molecules contribute to their persistence (Jiao et al., 2014, 2015; Arrieta et al., 2015). However, there are debates on the relative contributions of these two distinct mechanisms to the formation of the huge RDOC pool in the ocean (Arrieta et al., 2015; Zark et al., 2017; Wang et al., 2018; Shen and Benner, 2019). These debates advance studies examining the marine DOC molecular composition (Petras et al., 2017; Zark et al., 2017). The MCP generates both structural recalcitrance and a huge molecular diversity of DOC compounds each present at picomolar or subpicomolar concentrations (i.e., below microbial uptake thresholds) to evade being further consumed (Mentges et al., 2017; Jiao et al., 2018; Zark and Dittmar, 2018; Noriega-Ortega et al., 2019).

Microorganisms shape the marine ecosystems and drive the biogeochemical cycles (Azam et al., 1983; Azam and Malfatti, 2007; Falkowski et al., 2008). The carbon sequestration efficiency of both BCP and MCP is mainly regulated by microbial communities (Dang and Lovell, 2016; Zhang et al., 2018). POC and dissolved organic carbon (DOC) are the two distinct forms of organic carbon in the ocean, supporting distinct carbon sequestration processes, respectively. The formulation of the BCP concept started more than 35 years ago (Volk and Hoffert, 1985), and research on this front has been being highly active ever since (Siegel et al., 2016; Le Moigne, 2019). Although the concept of MCP is quite new, its research is gaining recognition and momentum (Zhang et al., 2018). In spite of the great research efforts, neither MCP nor BCP has achieved full understanding, regarding their respective quantitative contribution to climate modulation and the environmental and biological factors that may control their contributions and dynamics (Boyd, 2015; Robinson et al., 2018).

## Primary Production vs. Respiration

Organic matter provides the basis for the BCP and MCP to function. Most organic matter in the surface ocean is produced by primary producers. Thus, the marine primary production, which is subject to both top-down and bottom-up controls, is a key factor influencing BCP and MCP (Lechtenfeld et al., 2015; Siegel et al., 2016). Zooplankton grazing and viral lysis affect the composition, biomass, productivity, and partitioning of produced organic matter (particulate vs. dissolved) of the primary producer communities (Jiao et al., 2010; Sime-Ngando, 2014; Worden et al., 2015; Steinberg and Landry, 2017; Zimmerman et al., 2020). The availability and chemical speciation of nutrients play a critical role in determining the composition, abundance, and productivity of the marine photosynthetic microbial communities as well, and different phytoplankton may have distinct carbon export potentials (Herndl and Reinthaler, 2013; Richardson, 2019). Warming and nutrient scarcity may shift the phytoplankton communities, favoring taxa with small cell sizes, such as picocyanobacteria (Hutchins and Fu, 2017). *Prochlorococcus* are the most abundant and productive picocyanobacteria in oligotrophic oceans (Partensky et al., 1999; García-Fernández et al., 2004). They can hardly sink quickly enough on their own to directly contribute to BCP. However, this typical view is recently challenged (Richardson, 2019). *Prochlorococcus* are a potential source of transparent exopolymer particles (TEPs) that enhance marine aggregate formation and thus facilitate the BCP (Iuculano et al., 2017). Heterotrophic bacteria may also play a role in prompting TEP production and aggregate formation of *Prochlorococcus* (Cruz and Neuer, 2019). DOC released from *Prochlorococcus via* viral lysis and other processes may fuel the MCP for RDOC production (Zhao et al., 2017). The whole ecosystem structure has recently been proposed to majorly set the carbon sequestration efficiency (Guidi et al., 2016; Moriceau et al., 2018; Bach et al., 2019; Henson et al., 2019). These examples highlight the need of systematic and mechanistic investigations on the marine ecosystem key players and interactions, particularly in terms of their carbon sequestration roles and quantitative contributions.

Chemolithoautotrophic bacteria and archaea contribute organic carbon to the ocean as well (Herndl and Reinthaler, 2013; Dang and Chen, 2017). They not only play a key role in sustaining the chemosynthetic ecosystems related to deep-sea hydrothermal vents and cold seeps (McNichol et al., 2018; Dick, 2019) but also may contribute significantly to food web and energy transfer in non-extreme environments (Herndl and Reinthaler, 2013). Dark carbon fixation may provide substantial primary production in certain marine waters (Taylor et al., 2001; Yakimov et al., 2011; Celussi et al., 2017; Guerrero-Feijóo et al., 2018; La Cono et al., 2018). Microbial degradation and remineralization of marine particulate organic matter (POM) significantly lower the BCP efficiency (Turner, 2015; Dang and Lovell, 2016). However, the same processes regenerate nutrients and energy sources, likely playing a role in fueling chemolithoautotrophy and partially offsetting fixed carbon loss during sinking POM remineralization (Wright et al., 2012; Herndl and Reinthaler, 2013; Dang and Chen, 2017). Chemolithoautotrophy may help fuel the MCP as well. Ammonia-oxidizing archaea, usually dominant in mesopelagic and bathypelagic marine waters, release DOC to partially support *in situ* prokaryotic heterotrophy (Bayer et al., 2019). The contribution of chemolithoautotrophy to the global ocean's primary production, BCP, and MCP warrants further investigation.

Although the ocean's total primary production is very high (up to 50 Gt C/year), only a small fraction (<10%) is transported to the deep ocean *via* the BCP and even a smaller fraction (<1%) is sequestered for millennia (Henson et al., 2011; Bach et al., 2019; Fender et al., 2019; Giering et al., 2020). The majority of the marine primary production is converted back to CO_2_ in the ocean's twilight zone via community respiration, to which the microbes usually contribute the most (~50% to >90%) (Rivkin and Legendre, 2001; Sanders et al., 2016). Heterotrophic microbes uptake and respire DOC, and many particle-associated microbes secrete extracellular enzymes to hydrolyze POC into DOC (Arnosti, 2011; Orcutt et al., 2011; Dang and Lovell, 2016; Baltar, 2018). Respiration not only significantly lowers the BCP efficiency but also may cause deoxygenation and acidification in the affected marine waters (Cai et al., 2011; Oschlies et al., 2018; Robinson, 2019). Respiration may constrain the MCP as well (Robinson and Ramaiah, 2011; Dang and Jiao, 2014).

Although respiration is a fundamental metabolic process and the balance between respiration and primary production controls the ecosystem carbon storage capacity, respiration is much less investigated than primary production in the ocean (del Giorgio and Duarte, 2002; Arístegui et al., 2009; Robinson, 2019). This situation hinders our understanding of the ocean's carbon cycle. For example, the subtropical gyres cover ~40% of the Earth surface (Karl and Church, 2014). However, whether these oligotrophic open oceans are overall autotrophic (i.e., net CO_2_ sinks) or heterotrophic (i.e., net CO_2_ sources) is still being debated (Duarte et al., 2013; Ducklow and Doney, 2013; Williams et al., 2013; Koeve and Kähler, 2016), let alone confident prediction of their climate modulation potentials.

## Perspectives

The BCP (~0.2–0.5 Gt C/year) and MCP (~0.2 Gt C/year) may make similar contributions to long-term organic carbon sequestration (Guidi et al., 2015; Legendre et al., 2015; Giering et al., 2020), and both show climate geoengineering potentials (Le Moigne, 2019; Richardson, 2019). However, the BCP export efficiency has reduced ~1.5% over the past 33 years of climate warming (Cael et al., 2017), and warmer conditions will induce larger reductions (Boyd, 2015; Barange et al., 2017). The negative response of BCP to warming constitutes a positive feedback on climate change. The response of the MCP to climate change is currently not clearly known. Under the impacts of climate change and other anthropogenic perturbations, the global nitrogen cycle and ecosystem biodiversity may have already crossed the safe planetary boundaries (Rockström et al., 2009), exerting further negative impacts on the carbon cycle and climate through disrupting the coupled biogeochemical cycles (Schlesinger et al., 2011; Boyd et al., 2015). Research on fundamental processes and mechanisms of the BCP and MCP under varying oceanographic and climatic conditions is urgently needed, with a particular focus on integrating the major biogeochemical cycles and the ocean's biological, chemical, and physical processes for a better understanding of the marine carbon cycle and its response to climate change (Lucas et al., 2016; Hwang et al., 2017; Bif et al., 2018; Igarza et al., 2019; Quigley et al., 2019; Romera-Castillo et al., 2019). Mechanistic insights and implementation strategies have recently been proposed (Robinson et al., 2018; Yoon et al., 2018; Zhang et al., 2018; Boyd et al., 2019). Advances in upcoming marine carbon cycling research may also help overcome the uncertainty and difficulty in developing environmental-friendly ocean geoengineering techniques for climate change mitigation, the success of which may as well require interdisciplinary collaborations, strategic planning, technique innovations, and systematic investigations including both BCP and MCP for integrated ocean carbon sequestration enhancement (Polimene et al., 2018; Emerson, 2019; Sloyan et al., 2019; Sogin et al., 2019; Zhang et al., 2019).

## Author Contributions

The author confirms being the sole contributor of this work and has approved it for publication.

## Conflict of Interest

The author declares that the research was conducted in the absence of any commercial or financial relationships that could be construed as a potential conflict of interest.
